# First Nationwide Surveillance of *Culex pipiens* Complex and *Culex torrentium* Mosquitoes Demonstrated the Presence of *Culex pipiens* Biotype *pipiens*/*molestus* Hybrids in Germany

**DOI:** 10.1371/journal.pone.0071832

**Published:** 2013-09-11

**Authors:** Martin Rudolf, Christina Czajka, Jessica Börstler, Christian Melaun, Hanna Jöst, Heidrun von Thien, Marlis Badusche, Norbert Becker, Jonas Schmidt-Chanasit, Andreas Krüger, Egbert Tannich, Stefanie Becker

**Affiliations:** 1 Bernhard Nocht Institute for Tropical Medicine Hamburg; Molecular Entomology Group, Hamburg, Germany; 2 Bernhard Nocht Institute for Tropical Medicine Hamburg; Department of Molecular Parasitology, Hamburg, Germany; 3 Bernhard Nocht Institute for Tropical Medicine Hamburg; WHO Collaborating Centre for Arbovirus and Haemorrhagic Fever Reference and Research, Hamburg, Germany; 4 Biodiversity and Climate Research Centre Medical Biodiversity and Parasitology, Frankfurt am Main, Germany; 5 KABS e.V., Waldsee, Germany; 6 Bundeswehr Hospital Hamburg; Department Tropical Medicine; Entomology Group, Hamburg, Germany; 7 German Centre for Infection Research (DZIF), partner site Hamburg-Luebeck-Borstel, Hamburg, Germany; University of Liverpool, United Kingdom

## Abstract

Mosquitoes and other arthropods may transmit medically important pathogens, in particular viruses such as West Nile virus. The presence of suitable hosts and competent vectors for those zoonotic viruses is essential for an enzootic transmission, which is a prerequisite for epidemics. To establish reliable risk projections, it is an urgent need for an exact identification of mosquito species, which is especially challenging in the case of sibling species, such as *Culex. pipiens pipiens* biotypes *pipiens* and *molestus*. To facilitate detection of different *Culex pipiens* forms and their hybrids we established a multiplex real-time PCR. *Culex pipiens* samples were obtained by egg raft collection and rearing until imago stage or adult sampling using CO_2_ baited traps and gravid traps. In total, we tested more than 16,500 samples collected all over Germany in the years 2011 and 2012. The predominant species in Germany are *Culex pipiens pipiens* biotype *pipiens* and *Culex. torrentium*, but we also detected *Culex pipiens pipiens* biotype *molestus* and hybrids of the two *pipiens* biotypes at sites where both species occur sympatrically. This report of a potentially important bridge vector for West Nile virus might have major impact in the risk projections for West Nile virus in Germany.

## Introduction

In the past decades, West Nile virus (WNV) has spread from Africa and conquered various regions of temperate climate with substantial outbreaks in North America and Europe. WNV belongs to the group of arthropod-borne viruses (arbovirus), and is transmitted by mosquitoes. Accordingly, the spread of WNV is dependent on the presence of suitable mosquito vectors, and knowledge of the local mosquito species and their distribution are prerequisites for regional risk assessments of possible arbovirus-outbreaks. In Europe and other temperate regions, members of the *Culex pipiens* complex are the most ubiquitous mosquito species [1], [2] which serve as principal vectors for various arboviruses including WNV [3], [4], [5], [6], [7]. *Culex pipiens pipiens* (Linnaeus 1758) (*Cpp.*) can be subdivided into two distinct biotypes, which are morphologically indistinguishable but differ greatly in physiology and behaviour. The biotype *pipiens* is mainly ornithophilic (i.e. bird biting host preference), anautogenous (requires blood meal for first oviposition) and eurygamous (mating in outdoor swarms only), whereas the biotype *molestus* (Forskål 1775) is mammophilic (i.e. prefers biting mammals incl. humans), autogenous (first oviposition without prior blood meal) and stenogamous (ability to mate in a narrow space) [1]. In addition, *Culex torrentium*, another *Culex* species of temperate regions, exhibits virtually the same bionomic and morphological characters as *Cpp.* biotype *pipiens*. Although *Cpp.* and *Cx. torrentium* can be differentiated by the morphology of the male hypopygia, wild-caught females of both species with a spoiled thoracic chaetotaxy (presence of specific setae and/or scales) are morphologically indistinguishable. Presently, there is ongoing discussion whether they belong to different subgroups within the *pipiens* group of the subgenus *Culex* [8], or whether they should be treated as sibling species of the *pipiens* complex [9]. Another isssue of importance for WNV risk assessment is the identification of hybrids between *Cpp. pipiens* and *molestus* biotypes. These hybrids show opportunistic feeding behaviour and may serve as important bridge vectors for the transmission of viruses from infected birds to humans as revealed during outbreaks in the United States [10]. So far, hybrids of *pipiens* and *molestus* biotypes have been occasionally observed in several parts of Europe, but have not been described for Germany [11], [12]. However, information is lacking for most European countries about the distribution and composition of the different *Culex* species, biotypes or biotype hybrids due to the lack of suitable protocols that allow sensitive and specific high throughput screening of large sample sizes, generated during nationwide mosquito surveys. Here we report on a newly developed multiplex real-time PCR that allows discrimination of the various morphologically indistinguishable *Culex* species and biotypes. The method was used to analyse more than 16,500 *Culex* specimens from a recent nationwide mosquito surveillance program in Germany. The results indicate an uneven distribution of *Cpp*. and *Cx. torrentium* throughout the country, sympatric occurrence of *Cpp.* biotype *pipiens* and biotype *molestus* and first detection of *Cpp.* biotype *pipiens* and biotype *molestus* hybrids at different locations in Germany.

## Materials and Methods

### Mosquito samples and morphological species identification

Mosquitoes were trapped with CO_2_ baited traps or gravid traps from May to September 2011 and 2012 (Table 1). In addition, mosquito eggs were collected from suitable breeding sites such as water ponds or rain barrels. Two strategies were used to obtain *a priori* identified reference samples of the different taxa: Firstly, in the case of the morphospecies *Cpp.* and *Cx. torrentium* single egg batches collected outdoors were reared and the resulting adult males hypopygia were assessed [9] to determine the identity of each egg batch. Secondly, the identity of *Cpp.* biotype *molestus* was inferred from the behaviour of wild-caught and colonised F0 females and their offspring. For instance F1 females, produced by F0 females (collected indoors), are characterized by immediate stenogamy and autogeny without adaptation or selection over several generations. Another character for distinguishing biotype *molestus* was the shape of the egg raft, which is smaller and more irregular than the boat-like rafts of biotype *pipiens* and *Cx. torrentium*. As a positive control for the *molestus*×*pipiens* hybrid detection we used the F1 progeny of a *Cpp.* biotype *pipiens*×*Cpp.* biotype *molestus* experimental cross. The *Cx. p. quinquefasciatus* laboratory culture was obtained from Bayer HealthCare (Bayer; Germany).

**Table 1:** Trapping-sites of the nationwide surveillance program selected for the study.

Trapping-site	Coordinates	Trap type	Number of individuals (number of pools)
**Upper Rhine valley/Baden-Württemberg**			
Neckargerach	N 49°23′	GT	93 (8)
	E 9°4′		
Heidelberg	N 49°24′	GT	3800 (162)
	E 08°39′		
Heidelberg	N 49°24′	GT	977 (85)
	E 08°39′		
Karlsruhe - Island Rott	N 49°09′	EVS	43 (2)
	E 08°23′		
Karlsruhe - Russheim	N 49°11′	EVS	203 (11)
	E 08°25′		
Mümling-Grumbach	N 49°46′	GT	106 (9)
	E 8°59′		
Weinheim 2011	N 49°31′	GT	414 (19)
	E 08°38′		
Weinheim 2012	N 49°31′	GT	2002 (112)
	E 08°38′		
Beerfelden	N 49°33′	GT	33 (5)
	E 8°57′		
Eberstadt	N 49°48′	GT	194 (11)
	E 8°38′		
Großsachsen	N 49°31′	GT	418 (18)
2011	E 08°40′		
Großsachsen	N 49°31′	GT	558 (31)
	E 08°40′		
Hemsbach	N 49°35′	GT	77 (9)
	E 8°39′		
Waghäusel	N 49°15′	EVS	139 (8)
	E 08°31′		
Sandhausen	N 49°20′	GT	265 (13)
	E 8°39′		
**Lower-Rhine Valley/Palatine**			
Bad Dürkheim	N 49°22′	GT	52 (28)
	E 08°08′		
Bobenheim-Roxheim	N 49°34′	GT	222 (26)
	E 8°21′		
Dirmstein	N 49°34′	GT	549 (134)
	E 08°14′		
Kühkopf 2012	N 49°49′	EVS	36 (2)
	E 08°24′		
Kühkopf 2011	N 49°49′	EVS	197 (25)
	E 08°24′		
Neustadt a. d. Weinstraße	N 49°22′	GT	238 (74)
	E 08°08′		
Alsheim	N 49°45′	GT	1
	E 08°18′		
Mettenheim	N 49°44′	GT	182 (10)
	E 8°19′		
Römerberg	N 49°17′	GT	1721 (84)
	E 8°24′		
**Lake Constance/Bavaria West**			
Radolfzell (Lake Constance)	N 47°43′	EVS	144 (8)
	E 08°59′		
**Lake Chiemsee/Bavaria East**			
Chieming (Lake Chiemsee)	N 47°53′	EVS	89 (6)
	E 12°31′		
Hirschauer Bucht (Lake Chiemsee)	N 47°51′	EVS	13 (2)
	E 12°31′		
Chieming (Lake Chiemsee)	N 47°53′	EVS	6 (2)
	E 12°31′		
Plattling (Isar)	N 48°47′	EVS	83 (5)
	E 12°55′		
Stöttham (Lake Chiemsee)	N 47°45′	EVS	20 (2)
	E 12°31′		
Iffeldorf (Easter Lakes)	N 47°46′	EVS	1
	E 11°18′		
**Upper Elbe Valley/Saxonia**			
Coswig (Elbe)	N 51°51′	EVS	74 (11)
	E 12°26′		
**Oder Valley/Brandenburg**			
Oderaue (Oder)	N 52°47′	EVS	272 (52)
	E 14°14′		
**Baltic Sea/Mecklenburg**			
Greifswald - Eldena	N 54°05′	EVS	1960 (78)
	E 13°27′		
Greifswald - Loissin	N 54°07′	EVS	668 (27)
	E 13°30′		
**Metropolitan Region Hamburg**			
Langenlehsten	N 53°30′	Egg	349
	E 10°44	Collection	
Wulksfelde	N53°43′	BT	55
	E10°6′	GT	
HH/Ohlsdorf	N53°37′	BT	23
	E10°2′		
HH/Poppenbüttel	N53°39′	EVS	4
	E10°05′	BT	
HH/Cranz	N53°32′	ex- La.	8
	E09°46′		
HH/Hummelsbüttel	N53°38′	ex- La.	38
	E10°02′		
HH/Airport	N53°37′	BT	3
	E 10°00′		
HH/Barmbek	N53°35′	ex- La.	10
	E10°2′		
HH/Fuhlsbüttel	N53°37′	Swarm	12
	E10°01′		
HH/Ohlstedt	N53°41′	EVS	10
	E10°8′		
Höhbeck/Vietze 3	N53°03′	ex- Pu.	14
	E11°24′		
Stelle-Ashauen	N53°21′	BT	17
	E10°6′		
**Schleswig-Holstein**			
Sepel	54°07′	BT	4
	10°22′		
Gammendorf	N54°29′	BT	22
	E11°08′		
Warwerort	N54°08′	BT	67
	E08°55		
Wyk auf Föhr	N54°41′	indoors	10
	E8°33′		
**Hesse**			
Heubach1	N50°22′	ex- La.	24
	E 09°42′		
Heubach2	N50°22′	ex- La.	39
	E 09°42′		
Heubach3	N50°22′	ex- La.	7
	E 09°42′		
**total individuals**			**16566**

Trap types: BT: Biogents Sentinel; GT: Gravid trap; EVS: Enceph. Vector Surveillance Trap (Bioquip); ex-La.: reared from larvae ; ex-Pu.: reared from pupae.

With the exception of Hamburg Airport and Ohlsdorf all sampling sites within the various cluster areas are located either on public grounds for which specific permissions was not required, or on privately owned land. All owners of the private grounds gave permission to perform this study.

Permissions for Hamburg Airport and Ohlsdorf cemetery in Hamburg were given and are attached with manuscript submission.

The traps used in this study were specific for mosquitoes and did not catch any endangered or protected species.

### DNA extraction and multiplex real-time PCR assay

Mosquitoes collected at the various study sites were frozen at −70°C and transported to the laboratory where they were first identified morphologically at the genus level [13]. Subsequently, *Culex* ssp. from individual collections were pooled to up to 25 specimens per pool. All pooled samples were placed in sterile 2-mL cryovials, and subsequently maintained at −70°C until being assayed. Each mosquito pool was triturated in 500 µL of cell culture medium (high glucose Dulbecco's modified Eagle's medium (Sigma-Aldrich) with 10% heat-inactivated fetal bovine serum, 100 U/mL penicillin, 100 µg/mL streptomycin, and 2.5 µg/mL amphotericin B using two stainless steel beads (5 mm; Qiagen) in a TissueLyser (Qiagen) for 2 min at 50 oscillation/s. The suspensions were clarified by centrifugation (5,000× g for 1 min), and the supernatant was used for DNA extraction with AquaGenomic™-Solution (protocol for *Drosophila* samples, MultiTarget Pharmaceuticals) or QIAamp viral RNA mini kit according to the manufacturer's instructions.

The extracted DNA was analyzed by a newly designed multiplex real-time PCR using the primers for *Culex pipiens* F (5′- GCGGCCAAATATTGAGACTT -3′; nucleotide [nt] position 3 to 22 [the nt positions are given according to the numbering in the *Culex* reference strain 258c, GenBank accession number gb/DQ470148.1) and *Cx. pipiens* R (5′- CGTCCTCAAACATCCAGACA -3′; nt position 146 to 165) and probes *Cx. pipiens* all (5′- Cy55- GGAACATGTTGAGCTTCGGK -BBQ-1 -3′; nt position 77 to 95), *Cx. pipiens pipiens* biotype *pipiens* (5′- JOE GCTTCGGTGAAGGTTTGTGT-BHQ1 –3′) nt position 89 to 108 and *Cx. pipiens pipiens* biotype *molestus* (5′- Rox- TGAACCCTCCAGTAAGGTATCAACTAC- BHQ2 -3′; nt position 41 to 67; Reference strain 284b, GenBank accession number gb/470150.1) of the microsatelite locus CQ11. *Cx. torrentium* DNA was detected using the primers *Cx. torrentium* F (5′ -GACACAGGACGACAGAAA -3′; nt position 86 to 103), *Cx. torrentium* R (5′- GCCTACGCAACTACTAAA -3′; nt position 363 to 380) and the probe *Cx. torrentium* (5′- FAM- CGATGATGCCTGTGCTACCA-BHQ1 -3′; nt position 112 to 131) of the *ace2* gene (*Cx. torrentium* reference strain, GenBank accession number gb/AY497525.1). Multiplex real-time PCR was performed in a 20 µL reaction volume using HotStarTaq® Master Mix Kit according to the manufacturer's protocol (Qiagen). The specific primer molarities and sequence alignments of the loci used for primer design are shown in Figure S1.

## Results and Discussion

### Multiplex real-time PCR for simultaneous detection and differentiation of *Cpp.* biotypes and *Cx. torrentium*

**Figure 1:**
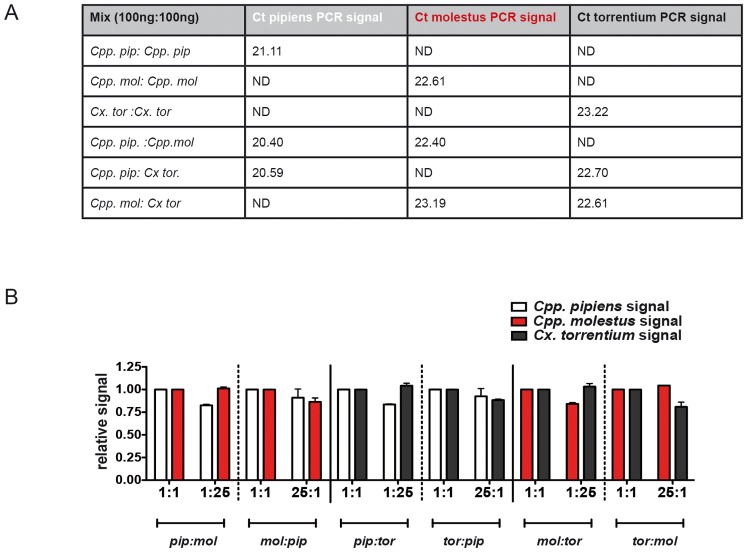
Establishment of a multiplex qPCR to differentiate *Cpp.* biotypes, biotype hybrids and *Cx. Torrentium.* A) Signal intensities for single species and mixed species DNA samples. The DNA of single individuals of the three taxa was extracted and quantified. For the reaction mixtures, either 200 ng of single species DNA templates or a 1∶1 mix of two species DNA templates (100 ng DNA species 1 and 100 ng species 2) were subjected to PCR testing. The reaction mix was prepared including the sets of primers and probes specific for *Cpp. pipiens*, *Cpp. molestus* and *Cx. torrentium* in a total reaction volume of 20 µL (for individual concentration please refer to Figure S1C). The signal intensities of each species-specific probe were measured for single taxon samples and taxon mixtures and are expressed as crossing points (Ct-values). A sample that is not targeted by the respective species-specific probe, is indicated as non detected (ND). B) As pools of up to 25 mosquitoes were analysed, the detection of individual DNA from *Cpp.* biotype *pipiens*, *Cpp.* biotype *molestus* or *Cx. torrentium in mixed samples were analysed by* the multiplex qPCR test. DNA of single individuals from the three taxa was extracted and quantified. The reaction mixtures were prepared with either 1∶1 mixed DNA template of two species (100 ng of each single species DNA sample) or 1∶25 diluted DNA sample (8 ng DNA of species 1 and 192 ng DNA of species 2). Subsequently, the DNA templates were subjected to a reaction mix containing species-specific sets of primer/probe for all three taxa and tested for amplification signals. The signal intensity for the 1∶1 mixture was set to 1 and signal intensities of 1∶25 mixed samples are expressed as relative values. The signal intensities are color-coded as followed: *Cpp. pipiens* in white, *Cpp. molestus* in red and *Cx. torrentium* in black. In the graph, relative signal intensities for 1∶25 DNA mixes of *Cpp.* biotype *pipiens* and *Cpp.* biotype *molestus* are named pip∶mol when *Cpp. pipiens* DNA was diluted in *Cpp. molestus* DNA, or mol∶pip when *Cpp. molestus* DNA was diluted in *Cpp. pipiens* DNA. Accordingly 1∶25 DNA mixes of *Cpp. pipiens* with *Cx. torrentium* were named pip∶tor if *Cpp. pipiens* DNA was diluted in *Cx. torrentium* DNA and tor∶pip if *Cx. torrentium* DNA was diluted in *Cpp. pipiens* DNA. The same nomenclature was used for 1∶25 mixes of *Cpp.* biotype *molestus* DNA with *Cx. torrentium* DNA. Values presented are the mean and standard deviation of two independent experiments.

Molecular assays to differentiate *Cpp.* and *Cx. torrentium* or to distinguish between the *Cpp.* biotypes reported so far are based on gel electrophoretic analyses of particular DNA fragments amplified by PCR [14], [15], [16]. These assays are time-consuming and prone to laboratory contamination, which are major drawbacks for the analysis of large sample sizes. To circumvent those problems we have developed a multiplex real-time PCR that allows differentiation of otherwise indistinguishable *Culex* mosquitoes in a single PCR reaction in a closed tube format. The assay targets the gene locus for acetylcholinesterase 2 (*ace2*) to discriminate between *Cx. torrentium* and *Cpp.* and the CQ11 microsatellite locus for discrimination between *Cpp.* biotypes [14], [16], [17]. Using a large collection of about 350 well-defined mosquito specimens (consisting of 227 *Cpp. pipiens*, 3 *Cpp. molestus* and 119 *Cx. torrentium* samples), the assay was evaluated and revealed 100% specificity for the respective *Culex* species or biotypes. Moreover, the PCR clearly identified hybrids generated by laboratory crosses between *Cpp.* biotype *pipiens* and biotype *molestus*. There was no or only little signal reduction when the assay was run with mixed DNA samples from the two *Cpp.* biotypes and *Cx. torrentium* as indicated by the similarity of Ct-values for mixed versus single DNA preparations (Figure 1A). As samples from surveillance programs are often analysed as pools of morphological identical mosquitoes, the multiplex real-time PCR was assessed using various numbers of *Culex* species or biotypes in mixed pools of up to 25 individuals. Although signal intensity was dependent on the number of insects to be detected, PCR specifically identified as little as single individuals in a mix of 25 mosquitoes (Figure 1B). Taken together, by combining simultaneous analyses of the two loci CQ11 and ace2, the multiplex real-time PCR reported here shows all characteristics required for large-scale analyses and differentiation of *Cpp.* biotypes *and Cx. torrentium*, respectively, in a single assay format.

### Distribution of *Cpp.* biotypes and *Cx. torrentium* in Germany

**Figure 2:**
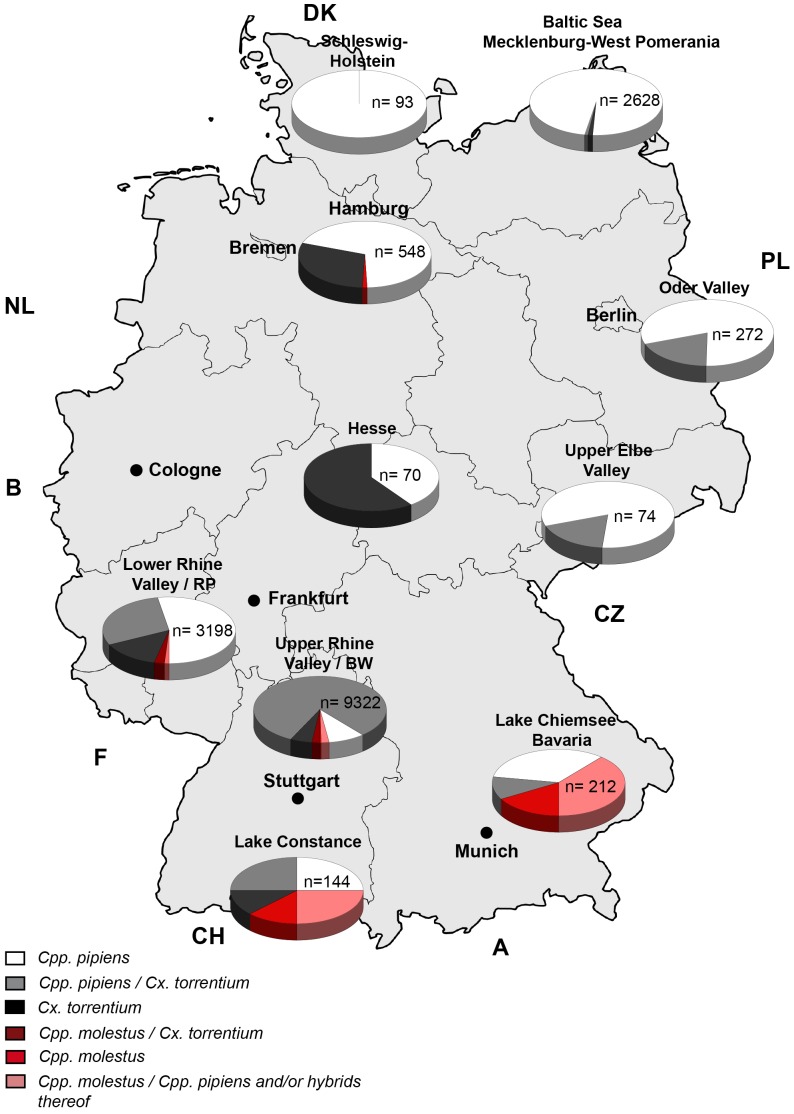
Classification of *Culex* samples from the German mosquito surveillance program. Graphical representation of the Culex species composition in Germany. 48 different trapping sites in Germany were combined according to their geographical relatedness to form 10 cluster areas shown representing Lower Rhine Valley and further sites in Palatine, Upper Rhine Valley and further sites in Baden-Württemberg, Lake Constance, Lake Chiemsee and other sites in Bavaria, Hesse, Upper Elbe Valley in Saxonia, Oder Valley in Brandenburg, Baltic Sea in Mecklenburg-West Pomerania, Metropolitan Region Hamburg and various sites in Schleswig-Holstein (for more details see also Table 1)). White (*Cpp. pipiens*), black (*Cx. torrentium*) and red (*Cpp. molestus*) quarters indicate pools that were composed of a single species. Grey (*Cpp. pipiens*+*Cx. torrentium*) and dark-red (*Cpp. molestus*+*Cx. torrentium*) quarters indicate pools composed of two species. With the current set-up (i.e.using pooled samples) the composition of pink quarters could be either two biotypes *Cpp. pipiens* and *Cpp. molestus* or hybrids of both biotypes. The n-numbers given in the graphs notify total numbers of individuals analysed in each cluster.

Morphologically indistinguishable *Cpp./Cx. torrentium* samples, collected in 2011 and 2012, during a nationwide mosquito surveillance programme were subjected to the newly developed multiplex real-time PCR in order to determine the distribution of *Cpp.* biotypes *and Cx. torrentium* across Germany. The 48 collection sites were selected according to the following criteria: known mosquito habitats e.g. extensive wetlands and presence of migratory birds (risk of imported WNV) and represent 10 major cluster areas, namely the Lower Rhine Valley and further sites in Palatine, the Upper Rhine Valley and further sites in Baden-Württemberg, Lake Constance, Lake Chiemsee and other sites in Bavaria, Hesse, the Upper Elbe Valley in Saxonia, the Oder Valley in Brandenburg, the Baltic Sea coast in Mecklenburg-West Pomerania, Metropolitan Region Hamburg and various sites in Schleswig-Holstein (Table 1). A total of more than 16,500 Culex ssp. were analysed out of which 716 represent individual specimens and 1,081 represent pooled samples. A pool usually contained between 5 and 25 mosquitoes from the same trap of a given time-point. Overall the predominant *Culex* species in Germany was found to be *Cpp*, with a mean abundance of 58% at the 48 selected sampling sites. However, considerable regional differences in species abundance and species composition were observed (Figure 2). Populations from coastal habitats in the North comprise almost exclusively *Cpp.* whereas *Cx. torrentium* is apparently absent in this region. In contrast, *Cx. torrentium* comprises up to 60% of the *Cpp./Cx. torrentium* populations and shows substantial overlaps with *Cpp* at all other sampling sites. Moreover, biotype analyses indicated absence of *Cpp. molestus* in all samples from North and East Germany, except Hamburg metropolitan area where a small proportion of the *Culex* population of around 1.3% consisted of *Cpp.* biotype *molestus*. On the other hand, up to 50% of the *Culex* populations at sampling sites in South and Southwest Germany were found to consist of *Cpp.* biotype *molestus*.

Evidently, the use of different traps needs to be taken into account when evaluating regional species differences. Gravid traps were used more frequently within the clusters Lower and Upper Rhine valley as well as in the Hamburg metropolitan area compared to other clusters. Therefore, additional analyses were performed restricted to *Culex* specimens collected by CO_2_ baited traps (EVS and BG) but excluding mosquitoes obtained by gravid traps or from reared egg batches. The results indicate some changes in the relative abundances of *Cpp* and *Cx. torrentium* at some areas, in particular in the cluster area Upper Rhine Valley. However, the general distribution of Cx. *torrentium* and *Cpp* across Germany was unaffected. Moreover, trapping rates of *Cpp.* biotype *molestus* were not altered at all using the alternative dataset (see Figure S2).

However, this study did not aim at a systematic analysis of *Cpp.* habitats and therefore, we can neither confirm nor reject previous data on the occurrence of *Cpp.* biotype *molestus*, such as underground breeding or their preference for urban habitats [9]. Further detailed studies are needed to highlight this topic.

Regarding *Cx. torrentium*, it appears that this species has spread considerably within Europe and Germany during the last 60 years. Until the 1950s *Cx. torrentium* was considered rare in Central and Western Europe, primarily colonizing higher altitudes [18], [19], [20]. In a monograph from 1969, presence of *Cx. torrentium* was reported only at four localities in central and southern Germany [13]. Careful analyses of previous and recent data have suggested that the apparent increase in abundance and distribution of *Cx. torrentium* during recent years was probably due to ecological adaptation and anthropogenic spread, rather than the result of an increased awareness of taxonomists who may have previously misidentified *Cx. torrentium* as *Cx. pipiens*. Nowadays, *Cx. torrentium* is widespread in Europe though the exact distribution limits remain to be determined [9].

### Screen for *Cpp.* biotype *pipiens* and biotype *molestus* hybrids in selected areas

**Figure 3:**
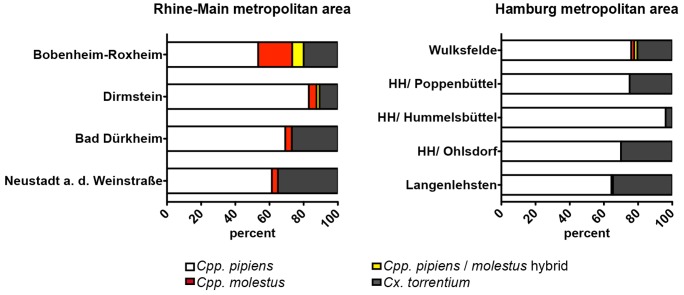
Identification of *Cpp.* biotype hybrids in two German metropolitan areas. Detailed species composition at the 4 sampling sites of the Rhine-Main metropolitan area (right graph) and 5 sampling sites at Hamburg metropolitan area (left graph). DNA samples from single individuals collected at these sampling sites were subjected to the multiplex real-time PCR and analysed for the presence of biotype hybrids. Bars represent species distribution in percent at each site. White indicates *Cpp.* biotype *pipiens*; red *Cpp.* biotype *molestus*; yellow hybrids of biotypes *pipiens* and *molestus*. Biotype hybrids were found at Dirmstein and Bobenheim-Roxheim trapping sites in Southwest Germany and at the Wulksfelde trapping site in northern Germany. Additionally, the presence of *Cx. torrentium* was assed and is indicated by the grey bars in both graphs.

In order to determine whether *Cpp.* biotype hybrids do exist in Germany a total of four sites within the Rhine-Main metropolitan region were selected where *Cpp.* biotype *pipiens* and biotype *molestus* were found to occur sympatrically. As pooled samples are not suitable for the analyses of possible hybrids, a number of individual mosquito specimens from the region were subjected to multiplex real-time PCR. Hybrids were detected at two of the four sites with a frequency of 1.7% and 6.6% respectively (Figure 3). Moreover, analysis of individual specimens from 5 sites of the Hamburg metropolitan area revealed another site positive for *Cpp.* biotype hybrids with a frequency of 1.8% (Figure 3). Given the relatively low prevalence of biotype *molestus* at the selected study sites (7.9% in average), the results suggest that crossbreeding between the two *Cpp.* biotypes is a frequent event in Germany. In fact, the incidence of biotype hybrids might be even higher than established by this limited analysis of individual mosquitoes, as overlapping occurrence of both biotypes was detected in the pooled samples of four southern cluster areas. Therefore, it is likely that at least some of the pools that revealed a signal for *Cpp.* biotype *pipiens* and *Cpp.* biotype *molestus* may contain biotype hybrids. Certainly, more comprehensive analyses are required, to determine the importance of this finding for arbovirus risk assessment in Germany. In particular, more individual mosquito samples need to be analysed to evaluate realistic hybrid frequencies and the vector competence of those hybrids for the transmission of WNV and other viruses. Nevertheless, the findings presented here are of concern in light of epidemic spread of WNV in the United States, where crossbreads of biotype *molestus* and *pipiens* frequently occurred and facilitated transmission of WNV to humans [10], [21].

## Supporting Information

Figure S1 **Sequence alignment of Ace-2 and CQ11 loci and detailed qPCR reaction mixture.** A) Sequence alignment for microsatellite locus CQ11 of *Cpp.* biotype *pipiens* (reference strain 258c; accession number gb/DQ470148.1) and *Cpp.* biotype *molestus* (reference strain 284b, accession number gb/470150.1). Primer binding sites for *Cpp. pipiens/molestus* forward and reverse primer are indicated in red, the control probe for *Cpp.* is indicated yellow, the species specific probes for *Cpp.* biotype *pipiens* and *Cpp.* biotype *molestus* are indicated in green and blue respectively. B) Sequence alignment for the Ace-2 locus of *Cpp.* biotype *pipiens* (reference strain isolate 41; accession number gb|JF430595.1) and *Cx. torrentium* (reference strain, accession number AY497525.1). The primer binding sites of *Cx. torrentium* forward and reverse are indicated in dark blue, the binding site of *Cx. torrentium* is indicated in orange. C) Detailed composition of the multiplex reaction mix used for all experiments presented in this publication. All primer and probes are colour-coded according to figure A) and B) and specific molarities in the 20 µL multiplex reaction are given.
(TIF)

Figure S2 **Classification of** ***Culex*** **samples from the German Surveillance program without gravid trap data.** Graphical representation of the species composition in Germany using the same dataset as figure 2 excluding all data derived from gravid traps. The 48 trapping sites in Germany were combined according to their geographical relatedness to form 10 cluster areas shown in the figure (Lower Rhine Valley and further sites in Palatine, Upper Rhine Valley and further sites in Baden-Württemberg, Lake Constance, Lake Chiemsee and other sites in Bavaria, Hesse, Upper Elbe Valley in Saxonia, Oder Valley in Brandenburg, Baltic Sea in Mecklenburg-West Pomerania, Metropolitan Region Hamburg and various sites in Schleswig-Holstein (see also table.1)). White (*Cpp. pipiens*), black (*Cx. torrentium*) and red (*Cpp. molestus*) quarters indicate pools that were composed of a single species. Grey (*Cpp. pipiens*+*Cx. torrentium*) and dark-red (*Cpp. molestus*+*Cx. torrentium*) quarters indicate pools composed of two species. With the current set-up (i.e. using pooled samples) the composition of pink quarters could be either two biotypes *Cpp. pipiens* and *Cpp. molestus* or hybrids of both biotypes. The n-numbers given in the graphs notify total numbers of individuals analysed in each cluster.
(TIF)
