# Supplementary figures and images for: First Nationwide Surveillance of Culex pipiens Complex and Culex torrentium Mosquitoes Demonstrated the Presence of Culex pipiens Biotype pipiens/molestus Hybrids in Germany
Source: PLoS One. 2013 Sep 11;8(9):e71832. doi: 10.1371/journal.pone.0071832 (PMC3770594; doi:10.1371/journal.pone.0071832)

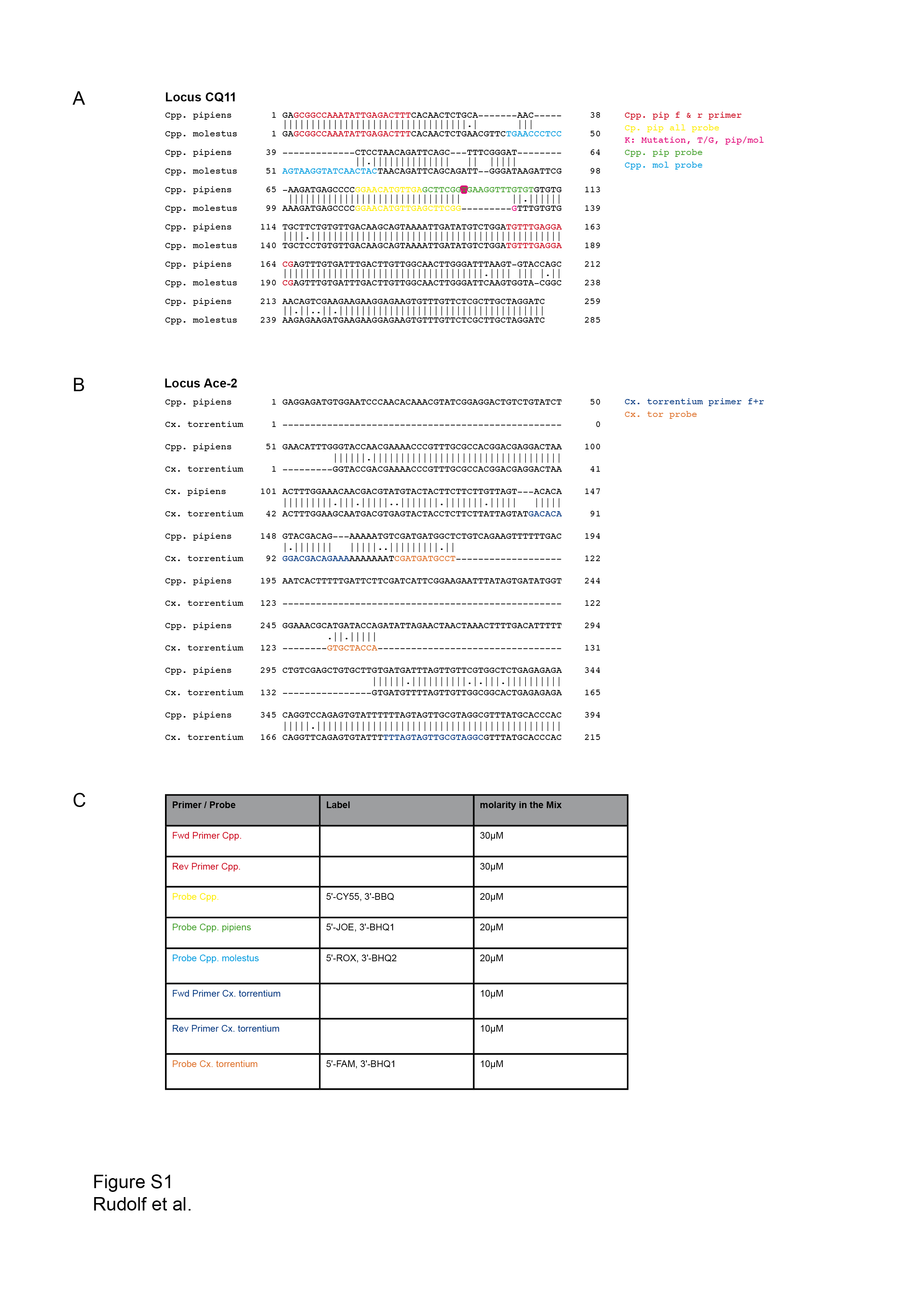

Supplement: Figure S1 — Sequence alignment of Ace-2 and CQ11 loci and detailed qPCR reaction mixture. A) Sequence alignment for microsatellite locus CQ11 of Cpp. biotype pipiens (reference strain 258c; accession number gb/DQ470148.1) and Cpp. biotype molestus (reference strain 284b, accession number gb/470150.1). Primer binding sites for Cpp. pipiens/molestus forward and reverse primer are indicated in red, the control probe for Cpp. is indicated yellow, the species specific probes for Cpp. biotype pipiens and Cpp. biotype molestus are indicated in green and blue respectively. B) Sequence alignment for the Ace-2 locus of Cpp. biotype pipiens (reference strain isolate 41; accession number gb|JF430595.1) and Cx. torrentium (reference strain, accession number AY497525.1). The primer binding sites of Cx. torrentium forward and reverse are indicated in dark blue, the binding site of Cx. torrentium is indicated in orange. C) Detailed composition of the multiplex reaction mix used for all experiments presented in this publication. All primer and probes are colour-coded according to figure A) and B) and specific molarities in the 20 µL multiplex reaction are given. (TIF) [file pone.0071832.s001.tif]

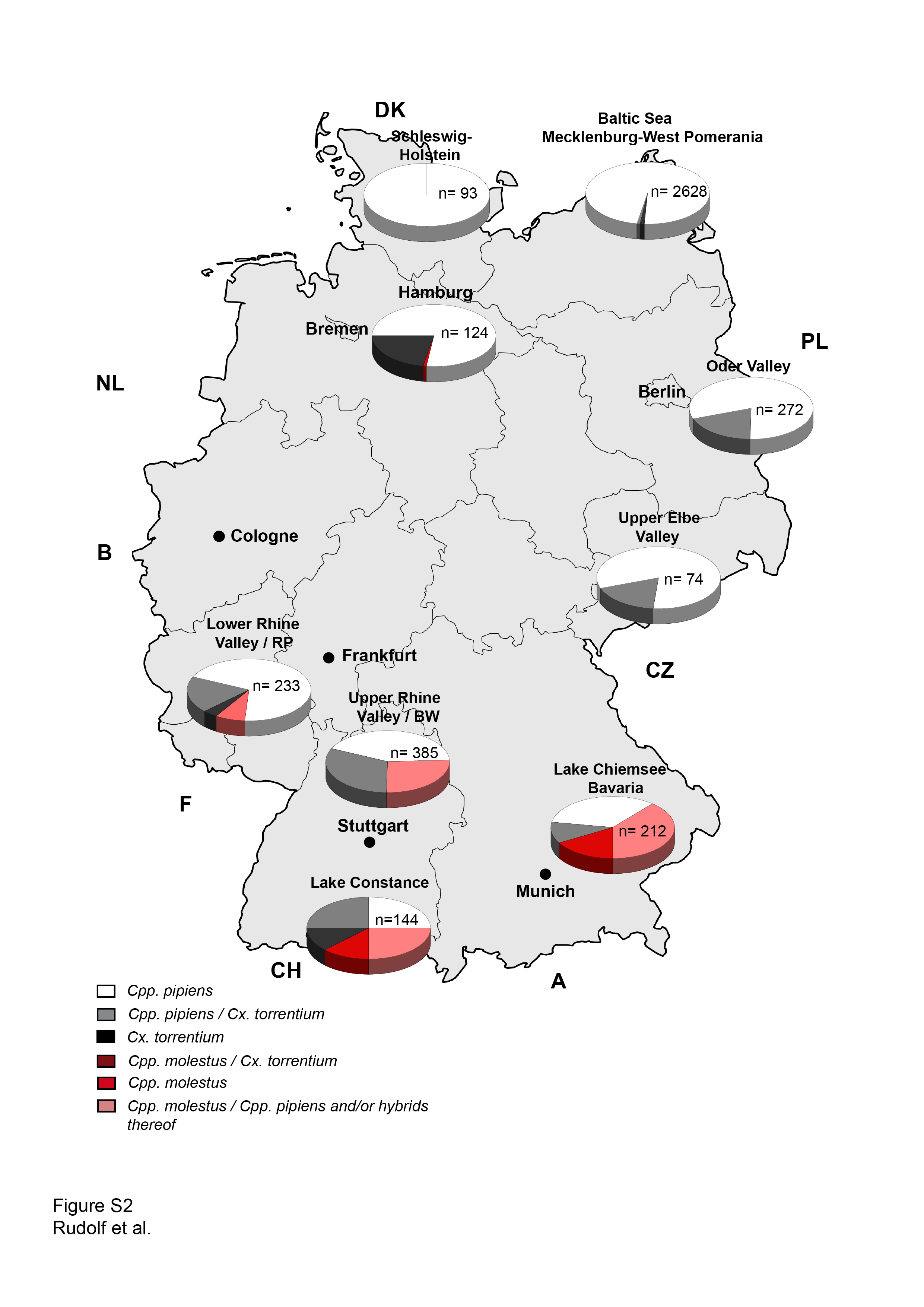

Supplement: Figure S2 — Classification of Culex samples from the German Surveillance program without gravid trap data. Graphical representation of the species composition in Germany using the same dataset as figure 2 excluding all data derived from gravid traps. The 48 trapping sites in Germany were combined according to their geographical relatedness to form 10 cluster areas shown in the figure (Lower Rhine Valley and further sites in Palatine, Upper Rhine Valley and further sites in Baden-Württemberg, Lake Constance, Lake Chiemsee and other sites in Bavaria, Hesse, Upper Elbe Valley in Saxonia, Oder Valley in Brandenburg, Baltic Sea in Mecklenburg-West Pomerania, Metropolitan Region Hamburg and various sites in Schleswig-Holstein (see also table.1)). White (Cpp. pipiens), black (Cx. torrentium) and red (Cpp. molestus) quarters indicate pools that were composed of a single species. Grey (Cpp. pipiens+Cx. torrentium) and dark-red (Cpp. molestus+Cx. torrentium) quarters indicate pools composed of two species. With the current set-up (i.e. using pooled samples) the composition of pink quarters could be either two biotypes Cpp. pipiens and Cpp. molestus or hybrids of both biotypes. The n-numbers given in the graphs notify total numbers of individuals analysed in each cluster. (TIF) [file pone.0071832.s002.tif]
